# Impact of a new conceptualized anti-reflux Trumpet stent on the quality of life of patients with advanced carcinoma of the cardio-oesophageal junction

**DOI:** 10.4314/gmj.v56i2.6

**Published:** 2022-06

**Authors:** Mahadevan D Tata, Nur Q A Mahazir, Ooi W Keat, Ismail A S Burud

**Affiliations:** 1 Department of Surgery, CMH Specialist Hospital, Seremban, 70200, Negeri Sembilan, Malaysia; 2 Department of Cardiothoracic Surgery, National Heart Institute (IJN), 50400, Kuala Lumpur, Malaysia; 3 Department of Surgery, Hospital Queen Elizabeth, 13a, Jalan Penampang, 88200 Kota Kinabalu, Sabah, Malaysia; 4 Department of Surgery, International Medical University, Clinical campus, Seremban, 70300, Negeri Sembilan, Malaysia

**Keywords:** Cardio-oesophageal Junction, cancer, palliation, stent, quality of life

## Abstract

**Objectives:**

To evaluate a newly developed, self-expandable anti-reflux Trumpet (ART) stent customized for cardio oesophageal junctional (COJ) cancer on the feasibility of deployment, stent migration, quality of life, and symptom relief

**Design:**

Prospective case series, Proof of concept pilot study

**Setting:**

Tertiary Health Care Center, Hospital Tuanku Jaafar, Seremban, Malaysia. Department of Surgery.

**Participants:**

A total of 17 patients diagnosed with advanced COJ tumour and who had never undergone any surgical, endoscopic, or chemoradiotherapy and indicated for stenting were recruited

**Interventions:**

The study period was over nine months, and follow-up was one-month post-stenting.

**Main outcome measures:**

Endpoint measures were feasibility of deployment of the new design, symptoms relief, early stent migration, early complication, GERD Q score, and (QOL)assessment.

**Results:**

The ART stent was inserted successfully in all cases (17/17, 100%). There were two stent migrations due to the flexibility of the stent at the neck. There were no early or post-stenting one-month complications associated with the procedure. A good flow of contrast was seen in all the stents deployed. GERD Q score was low in all patients pre and post-stenting. Post-stenting there was a relief of dysphagia, weight gain, and a 60% improvement in QOL score.

**Conclusions:**

ART stent is feasible and technically successful in COJ tumours. It provides good symptom relief, improves the QOL, and has minimal early complications.

**Funding:**

None declared

## Introduction

Cardio-oesophageal junction (COJ) carcinoma has the fastest growing incidence of cancer in the world. Despite recent medical advances, more than 80% of this cancer presents in advanced stages.^1^,^2^ The extremely aggressive behaviour and fast local and systemic dissemination of COJ carcinoma make it difficult to treat.^3^ They are associated with poor median survival and cause drastic deterioration in patients' performance status. This is mainly attributed to the inability to eat, causing poor nutritional status. The most common presenting symptom of COJ cancers is dysphagia which can be managed by inserting a Self-Expanding Metal Stent (SEMS) to allow oral feeding.

Gastrointestinal stents are one of the vastly explored treatment modalities and the combination of chemoradiotherapy for advanced COJ cancer in the current age. They are commonly used as a “bridge to surgery” or palliation to avoid surgery by maintaining the patency of the bowel lumen. Thus, this will increase nutrition intake and improve patients' quality of life (QOL).^4^,^5^ Many stents have been designed, created, and tested in recent years to minimize the complications of post-stent deployment, namely severe reflux, retrosternal chest pain, and stent migration, which are some of the common complications of an oesophagal stent occurring at a frequency of 7–75%.^4^,^5^,^6^,^7^

Despite the revolution in endoscopic enteric stents, most still display many shortfalls, such as migration, erosion, gastroenteric reflux disease, aspiration pneumonia, bleeding, and stent granulomas. Factors responsible for the complications related to the patients, surgeons, and the stent. Generally, oesophageal stents are associated with early complications which occur immediately or within 2–4 weeks post-procedure or are delayed.^8^

While many stents are designed for either oesophageal or stomach cancers, none are specifically designed for COJ tumours. Hence, there is a need to modify the design since COJ tumours pose different challenges for stents eg. unpredictable tumour growth, bleeding, angulation of stent in the stomach, and stent granuloma. Therefore, it is crucial to identify and design a stent that is not only effective but also ergonomic for both the patient and the doctor.

This study evaluated a newly developed, self-expandable ART stent customized for COJ cancer. Other endpoint measurements studied were the feasibility of the stent deployment by the endoscopist, post-stenting complications, assessment of the QOL, and symptoms relief post stenting

## Methods

An ART stent is a newly designed, anti-reflux stent with a flexible neck. The materials used are similar to the existing stents. Like other SEMS, ART stents (Trumpet stent; MI Tech. Korea) are composed of stainless steel, alloys such as elgiloy and nitinol, or a combination of nitinol and silicon. ART stent's structure is also made up of nitinol wire, an alloy of nickel and titanium, yielding increased flexibility that is helpful for stenting sharply angulated regions, namely the COJ at the cost of lesser radial force relative to stents made with other metals.

This study is a single-centre, open-label, proof of concept pilot study. This study was conducted according to good clinical practice and the principles of the Helsinki declaration after obtaining approval from the Medical Research and Ethics Committee, Ministry Of Health, Malaysia [NMRR-16-1318-31514]. Written informed consent was obtained from all patients per the Ministry of Health, Medical Research, and Ethics Committee guidelines. This study tested the feasibility of a new stent design, which was done in Hospital Tuanku Ja'afar from July 2017 until March 2018. A total of 17 patients who were diagnosed with advanced COJ tumour and indicated for SEMS insertion were recruited

Inclusion criteria were patients with advanced COJ cancer (stage IV) indicated for SEMS and medically fit. Patients excluded from the study were those with a history of surgery to the upper gastrointestinal tract, presence of mental disorders/ subnormal intelligence, skin or connective tissue disorders, and patients not fit for the endoscopic procedure All patients who fulfilled the criteria of having advanced adenocarcinoma of COJ and never underwent any surgical, endoscopic, or chemoradiotherapy were counselled and the endoscopist took consent. A single surgeon was assigned to deploy all the stents.

Olympus endoscopic system (EVIS EXERA III, OLYMPUS) was used for the stenting procedure. Monitored sedation was given throughout the procedure according to the local anaesthetic guideline for the general practitioner. Drugs used for sedation were intravenous Midazolam and intravenous Pethidine (according to anaesthetic guidelines during the endoscopic procedure). After inserting the stent, the patient was monitored for at least 2–4 hours. The patient was allowed to drink once fully conscious and was asked to follow the standardized-related diet plan one day after stenting. Patients were evaluated for post-stenting symptoms and the QOL. Post-procedure x-ray was done to look for stent placement on day-1 and day-30 post-insertion All patients were followed up periodically by phone and seen weekly in clinics to evaluate their progress. All patients were assessed pre and post stenting for GERD-Q score and QOL (EORTC QLQ C30 and EORTC QLQ OG25)

The primary endpoint assessed was: 1) the ease of deployment and manipulation of the stent. The secondary endpoints of the study were to evaluate the efficacy of ART stents in the relief of symptoms, for example, post-stenting nausea and epigastric pain, and the QOL post-stenting. The endpoints were measured and recorded using an endoscopist stenting code sheet, GERD-Q Questionnaire, and QOL questionnaire.

## Results

A total of 17 patients had undergone stenting. The mean age of these patients was 62.5years (48–81 years). All were advanced adenocarcinoma of COJ. Patients' demographics, the extent of obstruction, location, and histology are mentioned in Table 1. All patients had a successful deployment of the stent. There was no parachuting movement during stent adjustment and deployment. Stent migration was seen in the first two patients. After curved length adjustment for the flexible neck at the angle of HIS, there was no migration seen.

**Table 1:** Demographic and tumour characteristics of the patients

	n (%)
Mean Age	62.5
Tumor Location	
Cardia	16 (94.1)
Not involving cardia	1(5.9)
Mean duration of symptoms in months (Dysphagia)	2.05
Histopathology	
Moderately Differentiated Adenocarcinoma	1 (5.9)
Poorly Differentiated Adenocarcinoma	16 (94.1)
Obstruction	
Able to pass 9.9mm scope	7 (41.1)
Able to pass 5.5mm scope	9 (52.9)
Unable to pass scope	1 (5.9)

All patients had immediate relief of symptoms after the procedure. All patients tolerated oral feeding on the same day of the procedure. There was no change in the GERD Q score before and after the procedure. There was no worsening of the score at 30 days of follow-up. QOL score had a 60% improvement after stenting.

Median weight gain at four weeks was 4.37 kg. At 30 days after the procedure, there was no migration or dysphagia seen in any patient. (Table 2).

**Table 2:** Endpoint measurement related to the post-stenting outcomes

	n (%)
**Technical Success**	17(100)
**Dysphagia score after 4 weeks (Median)**	0 (0)
**Median weight gain within 4 weeks (Median)**	4.37kg
**Tissue growth within 4 weeks**	0 (0)
**Stent migration within 4 weeks**	2 (11.7)
**Food obstruction within 4 weeks**	0 (0)
**30-day mortality**	0 (0)

## Discussion

Currently, most of the SEMS used are either made for oesophagal or stomach cancers. SEMS is superior in terms of improving dysphagia dramatically. However, these SEMS have a few complications such as stent migration, stent granuloma, sludge accumulation, food impaction, epigastric pain, retrosternal pain, bleeding, perforation, etc. 7,9

This prompted the development of ART stent specific for COJ tumours. In the present study, the newly developed stent has shown 100% success in deployment. The first two cases had distal stent migration, due to a change in the curved measurement of the flexible neck length that occurred after deployment. This was corrected, hence after that, all other consecutive stents had no migration. In conventional stents, the measured and chosen lengths usually won't change after deployment as they extend 2–4 cms beyond each end.^10^ This is because conventional stents are measured along a straight line from the esophagogastric junction to the stomach. In the ART stent, the stent curves due to its flexible neck; hence the endoscopist must choose a stent 3cm longer before deploying to cater for stent bending after deployment.

This is a new revelation because no other available stent curves so much at the neck to preserve the angle of HIS. The stent fits at the COJ well and hence reduces symptoms.

**Figure 1:**
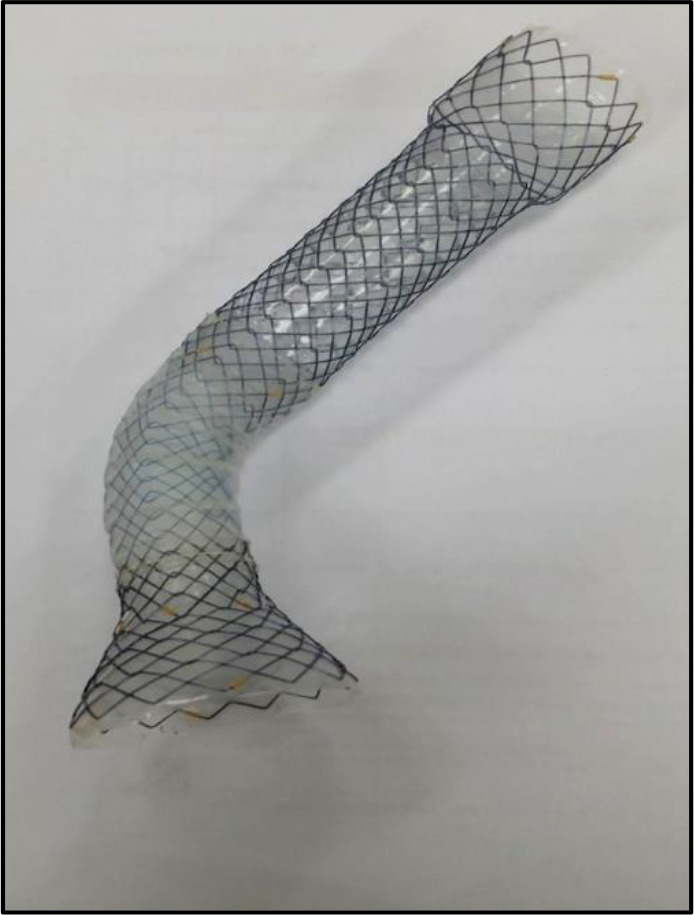
Anti-reflux trumpet stent with flexible Neck

**Figure 2:**
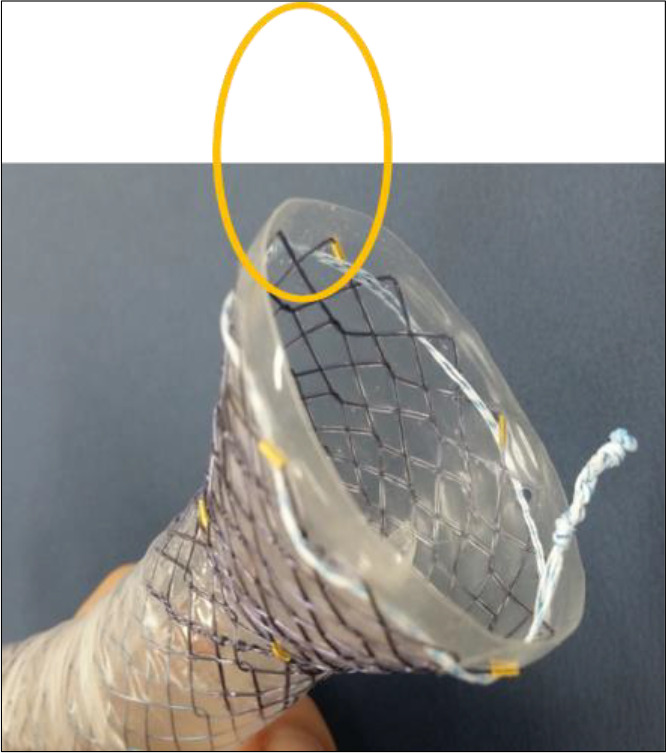
Distal end of the Anti-reflux Trumpet stent

**Figure 3:**
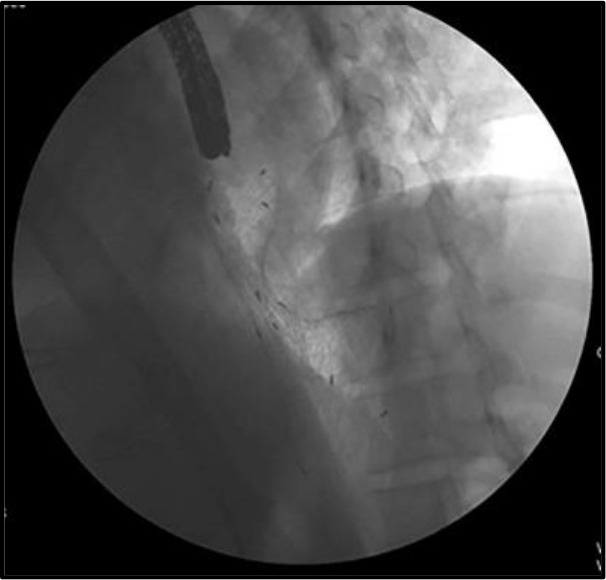
Distal flare with flexible neck fits well at the fundus

**Figure 4:**
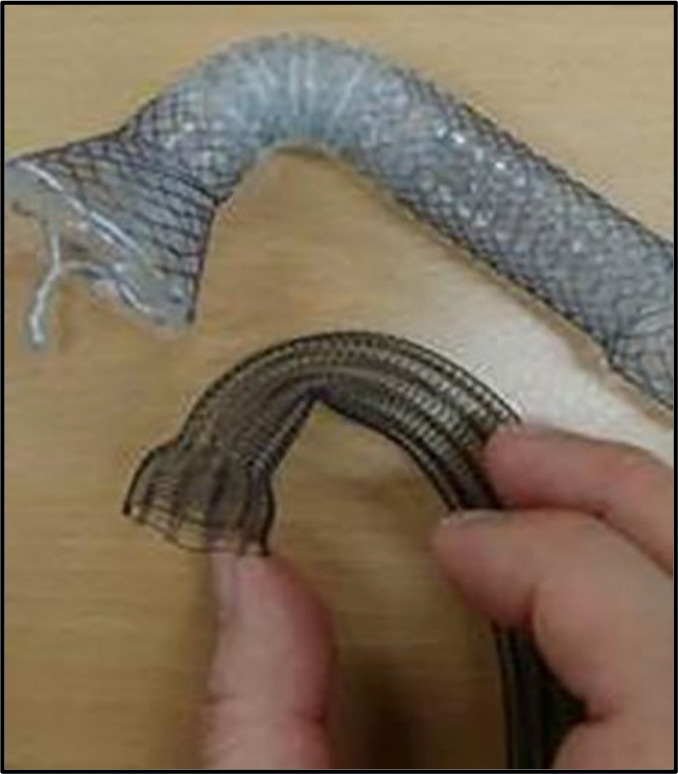
Flexible neck maintains pliability

The features of the ART stent are:

1) Flexible neck (Figure 1). This segment preserves the angle of HIS hence maintaining the physiologic anti-reflux anatomy of COJ. This prevents the stent from protruding to the anterior wall and does not cause epigastric pain when the patient lies supine;

2) This stent is made with a larger diameter as most patients with distal oesophageal obstruction will have a dilated oesophagus proximal to the tumour.

3) Large distal flare (Figure 2) was made to reduce the possibility of stent-related granuloma, and more silicon padding was added to give less mucosa irritation. A large distal flare was made to accommodate the large space of the fundus, and it does fit well in the fundus (Figure 3).

4) Flexible neck covered with silicone and not bonded with it; enables movement to accommodate the patient's posture. The flexible neck component is designed to maintain pliability and radial force even at an acute angle (Figure 4).

5) S-shaped anti-reflux component placed inside the stent and not protruding outside may cause partial obstruction when folded.

6) Wire diameters at the distal flare were increased to give firmness during diaphragmatic movement, hence maintaining the radial force. The stent is made from the same material as other approved stents in the market. Side effects due to base material were not anticipated.

The QOL score showed dramatic improvement immediately after the stenting, as these patients were able to eat on Day 2 after insertion of ART stent. Most of the patients had relief from dysphagia and could eat a normal diet. We had a 60% improvement in the QOL score. This positively impacted the weight gain on average of 4.37kg due to improved feeding.

Length selection and measurement are challenging as curved lengths to range from 2–4cm after deployment. Endoscopists need to be informed and trained that this stent is curved. Hence measurements made using an endoscope need adjustment to accommodate for post-stenting bending of the stent. Tumour length and tumour site influences the estimation of the curved length. Curved length adjustment needs to be learned and requires hands-on experience.

## Conclusion

ART stent is safe, feasible, and more ergonomically specific for COJ tumours with correct curved length adjustment. It improves patients' symptoms and QOL.
